# A Photovoltaic Power Prediction Method Based on Data-Driven Interval Construction Belief Rule Base

**DOI:** 10.3390/s26061957

**Published:** 2026-03-20

**Authors:** Lin Wang, Wenxin Xu, Ning Ma, Wei He, Wei Fu, Xiping Duan

**Affiliations:** 1 School of Computer Science and Information Engineering, Harbin Normal University, Harbin 150025, China; 2024300734@stu.hrbnu.edu.cn (L.W.); 2024300740@stu.hrbnu.edu.cn (W.X.); fullway@hrbnu.edu.cn (W.F.); xpduan_1999@126.com (X.D.); 2 Intelligent Laboratory for Teaching and Development of Future Teachers, Harbin Normal University, Harbin 150025, China

**Keywords:** belief rule base, data-driven, evidential reasoning rules, photovoltaic power prediction

## Abstract

Accurate prediction of photovoltaic (PV) power is crucial for ensuring grid stability. The belief rule base (BRB) is a rule-based expert system capable of effectively handling nonlinear causal relationships. Therefore, it can be applied to PV power prediction. In practical prediction scenarios, a high-quality initial model can produce more accurate predictions. However, obtaining sufficient expert knowledge to determine the structure and parameters of the BRB is usually difficult. To address this issue, a PV power prediction method is proposed based on a data-driven interval construction belief rule base (DD-IBRB), which reduces the reliance on expert knowledge during model construction. First, a fuzzy clustering algorithm is designed to construct reference intervals. Then, a Gaussian membership interval function (GIBM) strategy is proposed to initialize the belief degrees. Next, a representative point selection mechanism is designed within the reference intervals. Model inference is subsequently performed based on evidential reasoning (ER) rules. Finally, a multi-population evolution animated oat optimization with parameter constraints (MEAOO) is used to optimize the DD-IBRB model. Taking the PV power output as a case study, the mean squared error is 0.00056, indicating that the proposed DD-IBRB method can effectively complete modeling and obtain accurate prediction results.

## 1. Introduction

With the growing global demand for clean energy, photovoltaic (PV) power generation has been widely applied as an important renewable energy source [1]. Accurate prediction of PV power helps with power scheduling and supply–demand balance, preventing energy waste or shortages. However, irradiance, voltage, and ambient temperature in PV systems exhibit inherent uncertainties, which cause significant power fluctuations and bring challenges to grid security and dispatch [2,3]. Therefore, improving the prediction accuracy of PV power generation is of great practical importance [4].

PV power prediction methods can be classified into statistical models, physical models and hybrid approaches [5]. These models differ in modeling principles, data requirements, and applicable scenarios. The relevant studies and their problems are summarized in Table 1. They are elaborated below:

Statistical models do not require a deep understanding of the internal mechanisms of the PV system, focusing only on the input and output. Zhao et al. proposed a network architecture based on the VMD-KELM. The architecture was used to predict the power output of PV plants during severe weather events, effectively assessing the impact of adverse weather on power generation [6]. Fan et al. developed a coupled intelligent optimization prediction model. The model uses statistical techniques to reveal the uncertainty of PV systems and reduce prediction errors [7]. Wang et al. proposed an ultra-short-term spatiotemporal prediction model for distributed PV generation. The model uses the spatiotemporal characteristics of PV systems to achieve accurate output prediction at different locations [8]. Jia et al. established an adaptive weight allocation mechanism that integrates multi-source data for PV power prediction [9]. Although statistical models can achieve high prediction accuracy, they require large amounts of data for model construction and often lack interpretability [10,11].

Physical models describe the operating mechanisms of PV systems and can calculate their key design parameters. For example, Gao et al. proposed a physics-constrained deep learning framework [12]. The framework uses decomposition and feature fusion. It achieves learning generalization and accurate prediction when the data are limited. Yang et al. developed a short-term interval prediction strategy for PV power that is based on spatiotemporal correlation and multi-factor interval constraints. The method reconstructs meteorological data and improves the accuracy of power prediction [13]. Chen et al. proposed a spatiotemporal PV power nowcasting method with predictor preselection functionality, allowing rapid and precise predictor selection under varying conditions [14]. However, the prediction accuracy of physical models depends on precise meteorological data and complete information about PV cells. In practice, parameters may be incomplete, and weather forecasts may be inaccurate. As a result, the modeling often cannot reach the desired level [15].

The hybrid method refers to the combination of two different methods. Sara et al. developed an integrated prediction algorithm. The algorithm combines numerical weather prediction data, physical models, and artificial neural networks to enhance direct normal irradiance prediction [18]. Lu et al. proposed a real-time prediction method that combines phase space reconstruction, an improved gray wolf optimizer, and an LSTM network [19]. This method has a clear advantage in terms of prediction accuracy. The hybrid approach combines the interpretability of physical models with the prediction accuracy of statistical models. It extracts useful information from data and uses prior knowledge at the same time. This approach ensures both prediction accuracy and interpretability, providing better support for decision-makers.

To build a precise and reliable PV power prediction system, two key issues need to be addressed. First, PV power generation is affected by meteorological factors such as solar radiation and temperature, which introduce uncertainty. Therefore, the prediction model must be capable of handling uncertain information. Second, as an important energy source for the power grid, PV generation requires prediction results that are interpretable to support the efficient and reliable operation of the grid and to enhance grid operators’ trust in the model. The BRB effectively balances mechanistic interpretability and data-driven performance during the modeling process. The BRB was proposed by Yang et al. in 2006 as a rule-based modeling approach [20]. It is composed of the ER rules, fuzzy theory, and IF–THEN rules. Currently, BRBs have been widely applied in fields such as health condition assessment [21,22], lifetime prediction [23,24], fault diagnosis [25,26], and risk evaluation [27,28]. In the field of PV power prediction [16], the BRB model has strong nonlinear modeling capability, enabling it to effectively represent the detailed causal relationships between antecedent attributes and outcomes. This also makes it highly effective in handling uncertainty, providing decision-makers with more accurate and reliable prediction results.

However, in the BRB model, the total number of rules is determined by the Cartesian product of reference values. The number of BRB rules increases exponentially, which can cause a rule explosion problem [29]. To address this issue, He et al. proposed the IBRB model. The IBRB model processes input data into interval reference values [30]. It introduces interval addition in the modeling process to construct belief tables, thereby mitigating the combinatorial rule explosion. In existing studies, the IBRB model that was proposed has demonstrated significant advantages in handling multi-attribute decision-making problems, making it more suitable for practical engineering applications. However, when initially establishing an IBRB model, expert knowledge in the relevant field is still needed to define the reference intervals and belief degrees. In some engineering problems, sufficient expert knowledge may be unavailable, making the construction of an IBRB model challenging [17]. Data-driven methods extract the parameters needed to build the model from historical data. This facilitates the construction of the IBRB initial model and reduces reliance on expert knowledge [31]. For example, Zhou et al. use the K-means++ algorithm to construct reference intervals in student performance prediction, but the initial belief degrees are still derived from expert knowledge [32]. When professional knowledge is extremely limited, the model construction and performance of such IBRB methods are still affected. Therefore, to address this issue, this paper proposes a data-driven IBRB method for photovoltaic power prediction. Unlike existing methods, DD-IBRB starts from the model structure and achieves the automatic acquisition of the complete IBRB structure from raw data. This includes the construction of reference intervals and the generation of belief degrees, thereby significantly reducing the reliance on expert knowledge. This model can further enhance the modeling capability of the IBRB framework even when expert knowledge is insufficient.

This paper contributes as follows: (1) A DD-IBRB model is proposed to accurately predict PV power generation. The model can be automatically constructed via data mining techniques, even when expert knowledge is limited. (2) Data mining algorithms are designed for the selection of antecedent attribute reference intervals for each rule. They are also applied to determine the belief distributions and the representative points within each interval. (3) To improve the prediction accuracy of the model and ensure the rationality of parameter optimization, a multi-population evolution animated oat optimization with parameter constraints is proposed.

The remainder of this paper is organized as follows: Section 2 discusses the challenges in constructing PV power prediction models and introduces the proposed DD-IBRB model. Section 3 describes the construction processes of initial parameters. Section 4 presents the inference and optimization methods of the DD-IBRB model. Section 5 provides a case study on PV power prediction to validate the effectiveness of the proposed approach. Section 6 concludes the paper.

## 2. Problem Formulation and Construction of the DD-IBRB Model

Section 2.1 describes the problem of PV power output prediction, while Section 2.2 presents the construction of a PV power prediction model based on the DD-IBRB approach.

### 2.1. Problem Formulation

When constructing a PV power prediction model based on the DD-IBRB framework, the following three issues must be addressed:

Problem 1: How to obtain reasonable initial model parameters when expert knowledge is limited.

When constructing an IBRB model, it is necessary to define initial parameters such as the reference intervals of the antecedent attributes, I, and belief degrees, β. These parameters typically rely on expert knowledge. However, in practical engineering, expert knowledge may be insufficient. As a result, some initial parameters cannot be accurately determined, which affects the preliminary construction of the model. To address this issue, this paper analyzes historical data to automatically generate the required parameters. The process of constructing the initial parameters is as follows:

(1) I=E(x,ϖ)

(2) β=F(x,I,ϕ)

where x denotes the input data, E(⋅) is the function used to generate the reference intervals, and ϖ represents the set of parameters required for generating the reference intervals. F(⋅) is the function used to generate the belief degrees, and ϕ represents the set of parameters required for generating the belief degrees.

Problem 2: How to reasonably construct the inference process of the PV power prediction model to enable prediction via the DD-IBRB.

In existing IBRB models, multiple data points falling into the same reference interval are assigned identical activation weights. This makes it difficult to accurately reflect the differences in highly fluctuating or discontinuous data and reduces prediction accuracy. To address this issue, this study proposes a method for adaptively generating activation weights based on input data features. The method improves the accuracy of the inference process and enhances the model’s adaptability to complex data. The inference process is as follows:

(3) y=f(x,Ω)

where y is the PV power prediction result, f(⋅) denotes the inference function, and Ω is the set of model parameters, including I,β.

Problem 3: How to optimize the parameters while maintaining the validity of the initial model, thereby improving the prediction accuracy of the DD-IBRB. In the DD-IBRB model, in addition to reference intervals and belief degrees, rule weights and rule reliabilities also have an important impact on modeling accuracy. Therefore, a reasonable optimization process is required to adjust these parameters. The optimization process can be described as follows:

(4) $$\Omega_{best}=Optimize(x,y,\tau )$$

where $\Omega_{best}$ represents the set of optimized model parameters, Optimize(⋅) denotes the optimization function, and τ represents the set of parameters required for the optimization process.

### 2.2. DD-IBRB Model

**Figure 1:**
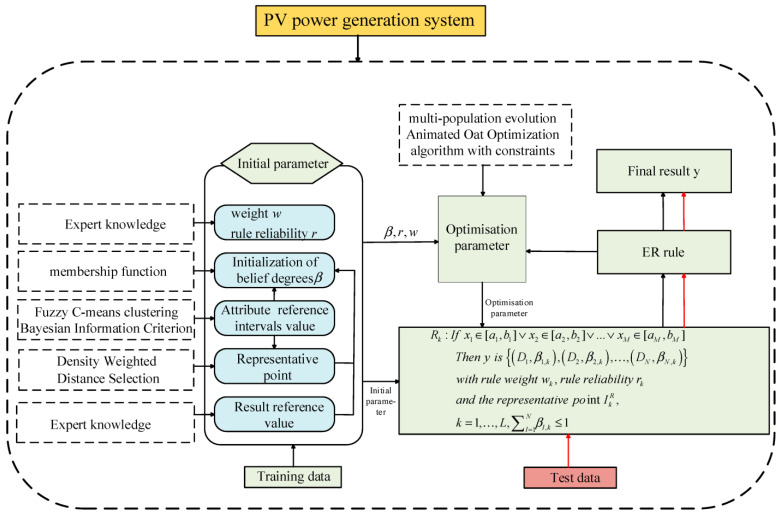
Overall structure of the DD-IBRB model.

The DD-IBRB consists of a series of belief rules. Assuming that the antecedent attributes are independent of each other, the kth rule of the DD-IBRB model can be expressed as follows:

(5) $$\begin{array}{l} R_{k}:If\;x_{1}\in [a_{1},b_{1}]\vee x_{2}\in [a_{2},b_{2}]\vee \ldots \vee x_{M}\in [a_{M},b_{M}]\\ \;\;Then\;y\;is\;\left\{\left(D_{1},\beta_{1,k}\right),\left(D_{2},\beta_{2,k}\right),\ldots ,\left(D_{N},\beta_{N,k}\right)\right\}\\ \;\;with\;rule\;weight\;w_{k},\;rule\;reliability\;r_{k}\\ \;\;and\;the\;representative\;po\mathrm{int}\;I_{k}^{R},\\ \;\;k=1,\ldots ,L,\sum_{l=1}^{N}\beta_{l,k}\leq 1 \end{array}$$

where $x_{i}(i=1,2,\ldots ,M)$ represents the antecedent attributes of the model, with M denoting the number of antecedent attributes. $\left\{\left(D_{1},\beta_{1,k}\right),\left(D_{2},\beta_{2,k}\right),\ldots \left(D_{N},\beta_{N,k}\right)\right\}$ denotes the belief distribution of the DD-IBRB output results. $D_{i}\left(i=1,\ldots ,N\right)$ represents the ith output grade, where N is the total number of output grades. $\beta_{i,k}\left(i=1,\ldots ,N\right)$ denotes the belief degree corresponding to the ith output grade. $\left[a_{i},b_{i}\right]$ represents the reference interval. $w_{k}\left(k=1,\ldots ,L\right)$ represents the weight of the rule, where L is the total number of rules. $r_{k}\left(k=1,\ldots ,L\right)$ denotes the reliability of the rule. $I_{k}^{R}$ represents the representative point of each reference interval, and is used to measure the degree to which the input data activate the corresponding rule. The structure of the PV power prediction model is shown in Figure 1.

## 3. Construction of Initial Parameters for the DD-IBRB Model

Section 3.1 introduces the construction process of the reference intervals. Section 3.2 presents the construction process of the representative points within each reference interval. Section 3.3 describes the construction process of the initial belief degrees.

### 3.1. Construction Process of the Reference Intervals

**Figure 2:**
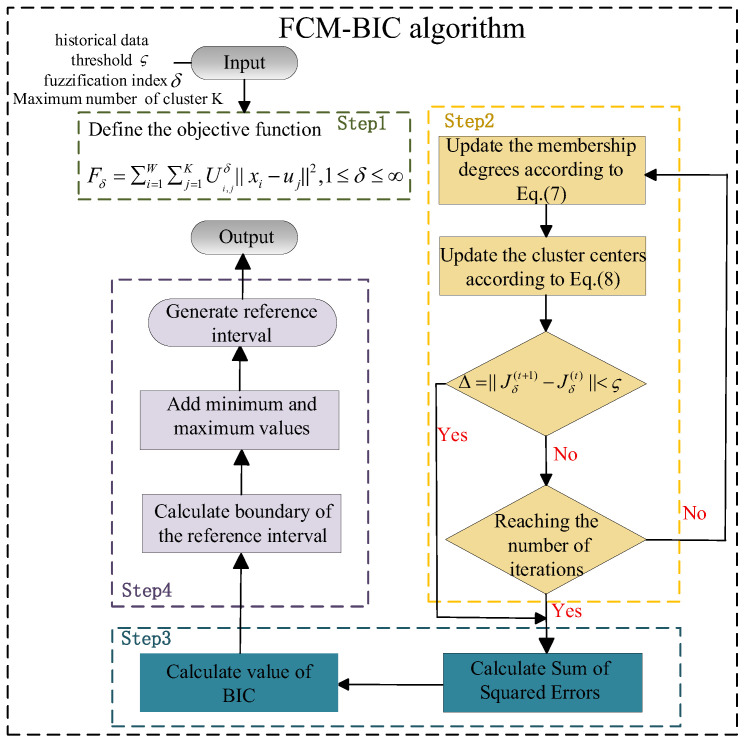
Structure of the FCM-BIC algorithm.

In the IBRB-based PV power prediction model, reference intervals play a fundamental role and are usually predefined by experts. However, when expert knowledge is insufficient or only historical data is available, constructing the model becomes challenging. To address this issue, an FCM-BIC method is proposed. In this method, fuzzy C-means (FCM) clustering is combined with the Bayesian information criterion (BIC) to automatically extract reference intervals for PV power. The algorithmic process is illustrated in Figure 2.

By analyzing historical data, typical values and distribution patterns of input variables can be identified [33]. Setting reference intervals in high-density regions can improve model adaptability and optimize inference accuracy. This study uses the FCM clustering algorithm, which calculates membership degrees and iteratively adjusts cluster centers to minimize the weighted squared distance, resulting in clusters that reflect the data characteristics [34]. To balance the number of reference intervals and model complexity, BIC is introduced as an evaluation metric. The optimal reference interval partitioning is determined by selecting the number of clusters with the smallest BIC value. The main steps of the FCM-BIC method are as follows:

Step 1: Define the objective function. In the IBRB-based PV power prediction, the proper setting of the number of reference intervals is crucial. If the number of intervals is too large, the rule base becomes redundant, and model complexity increases. If it is too small, the data characteristics may not be fully captured. Therefore, the maximum number of clusters K needs to be set according to the data distribution, with the reference intervals containing at least two values. The range of K is set to [2,K].

After determining the range of K, a clustering objective function is constructed to minimize the sum of squared Euclidean distances between the sample points and their corresponding cluster centers. The cluster centers are updated through iterative optimization until the objective function converges, resulting in the final clustering outcome. The objective function is defined as follows:

(6) $$F_{\delta }=\sum_{i=1}^{W}\sum_{j=1}^{K}U_{i,j}^{\delta }∥x_{i}-u_{j}{∥}^{2},1\leq \delta \leq \infty$$

where δ is the fuzzification index used to control the degree of fuzziness in the membership matrix, with a value greater than 1. In line with the systematic theoretical analysis and numerical experiments on FCM cluster validity by Pal et al. [35], we select δ=2, as it lies in the optimal interval [1.5, 2.5] for the fuzzification index and is the most widely accepted default value in FCM applications; W is the total number of train samples; $U_{i,j}$ denotes the membership degree of the ith data point belonging to the jth cluster; and $u_{j}$ represents the cluster center.

Step 2: Iteratively update the membership degrees and cluster centers. In each iteration, the membership degrees are updated based on the current cluster centers:

(7) $$U_{i,j}=\frac{1}{\sum_{k=1}^{K}{\left(\frac{||x_{i}-u_{j}|{|}^{2}}{||x_{i}-u_{k}|{|}^{2}}\right)}^{\frac{1}{\delta -1}}}$$

Then, the cluster centers are updated based on the newly calculated membership degrees:

(8) $$u_{j}=\frac{\sum_{j=1}^{W}{\left(U_{i,j}\right)}^{\delta }x_{i}}{\sum_{j=1}^{W}{\left(U_{i,j}\right)}^{\delta }}$$

This process is repeated until the change in the objective function $\Delta =∥J_{\delta }^{\left(t+1\right)}-J_{\delta }^{\left(t\right)}∥<\varsigma$ (where ς is a predefined threshold, we set ς=0.00001, which is the fixed threshold used in FCM experiments of Pal et al. [35] and is commonly used in FCM implementations because it provides a good balance between convergence accuracy and computational efficiency) or the maximum number of iterations T is reached. At this point, the iteration stops, and the obtained cluster centers represent the core aggregation regions of the data. Here, t denotes the current iteration number.

Step 3: Calculate the BIC. For each cluster number K, calculate the BIC.

(9) BIC=K⋅log(W)+W⋅log(SSE/W)

(10) $$SSE=\sum_{k=1}^{K}\sum_{j=1}^{W}U_{i,j}^{\delta }\cdot ∥x_{i}-u_{j}{∥}^{2}$$

A smaller BIC value indicates that the model achieves a lower fitting error with appropriate complexity. This leads to better overall clustering performance.

Step 4: Reference interval partitioning. On the basis of the optimal number of clusters $K_{optimal}$ and their corresponding cluster centers $\left\{u_{1},u_{2},\ldots ,u_{K_{optimal}}\right\}$, the reference intervals are calculated as follows:

(a) Calculate the midpoints between adjacent cluster centers as the interval boundaries:

(11) $$b_{j}=\frac{u_{j}+u_{j+1}}{2},\;\;\;j=1,2,\ldots ,K_{\mathrm{optimal}}-1$$

(b) Include the minimum and maximum values of the data to ensure that all input samples are covered:

(12) $$b=[Min(x),b_{1},b_{2},\ldots ,b_{K_{optimal}-1},Max(x)]$$

(c) Generate the set of reference intervals:

(13) $$I_{j}=[b_{j},b_{j+1}],j=1,2,\ldots ,K_{\mathrm{optimal}}$$

(14) $$I=[I_{1},I_{2},\ldots ,I_{K_{\mathrm{optimal}}}]$$

where $I_{j}$ denotes the jth reference interval, and where I represents the complete set of reference intervals.

### 3.2. Construction Process of Representative Points Within Reference Intervals

In the IBRB model, each reference interval corresponds to one rule. When the input data fall into a specific interval, the corresponding rule is activated. However, traditional methods assign the same activation weight to all data points within the same interval. This makes it difficult to capture subtle differences and reduces classification accuracy. For example, the values 3.1 and 4.9 both belong to the interval (3, 5) and receive the same weight, which may degrade model performance when the data distribution is uneven.

To address this problem, the DD-IBRB model introduces the concept of a representative point. The activation weights are dynamically calculated based on the matching degree between the input data and the representative point. This enables differentiated responses within each interval and improves the model’s adaptability to complex data. The representative point is determined by the density-weighted distance selection (DWDS) algorithm [36]. This algorithm takes into account both the distance between each data point and the cluster center, as well as the local density distribution. It selects points that are both geometrically representative and reflective of the data distribution. By balancing spatial distance and data density, DWDS allows the representative point to more accurately characterize the reference interval’s features. It overcomes the limitations of traditional geometric-center methods and significantly improves inference accuracy. The main steps of the DWDS method are as follows:

Step 1: Calculate Local Density. For each data point $x_{i}\in I_{j}$ within the interval $I_{j}$, compute its local density $\rho_{i}$, which measures the degree of concentration of that point within the interval.

(15) $$\rho_{i}=\underset{x_{k}\in I_{j}}{\sum }\exp\left(-\frac{\left|x_{i}-x_{k}\right|}{\gamma }\right)$$

where $x_{k}$ represents other data points within the interval, $\left|x_{i}-x_{k}\right|$ denotes the absolute distance between two points, and γ is set to 0.05 and controls the smoothness of the exponential kernel, thereby affecting the sensitivity of the density estimation.

Step 2: Calculate distance to the cluster center. For each data point $x_{i}\in I_{j}$ within the interval $I_{j}$, compute its absolute distance $V_{i}$ to the cluster center $u_{j}$.

(16) $$V_{i}=|x_{i}-u_{j}|$$

The smaller value of $V_{i}$ indicates that the data point is closer to the cluster center.

Step 3: Calculate the comprehensive score. The local density $\rho_{i}$ and the absolute distance $V_{i}$ are combined to calculate the overall score $S_{i}$ of each data point, and its suitability as a representative point is evaluated.

(17) $$S_{i}=\frac{\rho_{i}}{1+V_{i}}$$

Step 4: Select the representative point. Within the interval $I_{j}$, select the data point with the highest comprehensive score $S_{i}$ as the representative point.

(18) $$I_{k}^{R}=arg\max(S_{i})$$

### 3.3. Construction of Initial Belief Degrees

The IBRB model relies on expert experience to construct the initial belief rule table, which supports the inference process. The belief degrees provided by expert knowledge are critical for the transparency and credibility of the results. However, in practical applications, obtaining consistent expert knowledge is often difficult, leading to challenges in initializing the rule base.

To address this issue, this paper proposes an initialization method that combines both data-driven approaches and expert knowledge. Based on the preliminary result grade distribution provided by experts, the Gaussian membership interval function is introduced. The Gaussian membership interval function can smoothly and continuously represent the uncertainty between levels. Its probabilistic characteristics are highly consistent with the normalization constraints of confidence initialization and can naturally reflect low-probability deviation events. It aligns with the inherent requirements of confidence rule-base initialization, making it more suitable for the confidence initialization stage. The combined application of the two not only retains the guiding role of expert experience but also fully utilizes the statistical laws implied in the data, laying a more scientific foundation for subsequent model reasoning. The specific steps of this method are as follows:

Step 1: Determine which interval $x_{i}$ falls into.

(19) $$k=\{j|x_{i}\geq I_{j}(j,1)\;and\;x_{i}\leq I_{j}(j,2)\}$$

where $I_{j}(j,1)$ represents the lower bound of the jth interval, $I_{j}(j,2)$ represents the upper bound of the jth interval, and k represents the rule activated by $x_{i}$.

Step 2: Rule matching degree calculation. For each activated rule k, the interval boundaries $a_{j}=I_{j}(j,1)$ and $b_{j}=I_{j}(j,2)$ are extracted, and the representative point $I_{j}^{R}$ of the interval is taken. The membership relationship between $x_{i}$ and the rule within the interval is derived via the following representative point:

(20) $$h_{k}(x_{i})=\exp\left(-\frac{{\left(x_{i}-I_{j}^{R}\right)}^{2}}{2\sigma_{j}^{2}}\right)$$

(21) $$\sigma_{j}=\frac{b_{j}-a_{j}}{4}$$

where $\sigma_{j}$ represents the standard deviation of the reference interval $\left[a_{j},b_{j}\right]$.

Boundary Special Handling: If $x_{i}$ falls exactly on the right boundary $b_{j}$ of the interval and a subsequent interval exists, the matching degrees with the next interval $\left[a_{j+1},b_{j+1}\right]$ and its representative point $I_{j+1}^{R}$ are also calculated, and their weighted average is taken as:

(22) $$h_{k}(x_{i})=0.5(h_{k}(x_{i})+\exp\left(-\frac{{\left(x_{i}-I_{j+1}^{R}\right)}^{2}}{2\sigma_{j+1}^{2}}\right))$$

If $x_{i}$ falls exactly on the left boundary $a_{j}$ of an interval and a previous interval exists, the matching degrees with the previous interval $\left[a_{j-1},b_{j-1}\right]$ and its representative point $I_{j-1}^{R}$ are also calculated, and their weighted average is taken as follows:

(23) $$h_{k}(x_{i})=0.5(h_{k}(x_{i})+\exp\left(-\frac{{\left(x_{i}-I_{j-1}^{R}\right)}^{2}}{2\sigma_{j-1}^{2}}\right))$$

Step 3: Matching Degree Calculation for Output Results. For the sample output value $y_{i}$, its membership degree to the output reference value $D_{i}\left(i=1,\ldots ,N\right)$ is given by:

(24) $$\ell_{l,k}(y_{i})=\left\{\begin{array}{ll} \frac{D_{ο+1}-y_{i}}{D_{ο+1}-D_{ο}} & l=ο,D_{ο}\leq y_{i}\leq D_{ο+1}\\ \frac{y_{i}-D_{ο}}{D_{ο+1}-D_{ο}} & l=ο+1\\ 0 & otherwise \end{array}\right.$$

Step 4: Belief degree update. On the basis of the sample $x_{i}$, the membership degree is mapped to the corresponding result belief degrees. For each sample $\left(x_{i},y_{i}\right)$, traverse all its activated rules to update the belief degree matrix.

(25) $$\beta_{l,k}\leftarrow \beta_{l,k}+h_{k}(x_{i})\times \ell_{l,k}(y_{i})$$

Step 5: Normalization. Since the belief distribution is essentially a probability distribution, the belief degrees of all result categories within each rule must satisfy the constraint that their sum equals 1.

(26) $$\beta_{l,k}=\frac{\beta_{l,k}}{\sum_{l=1}^{N}\beta_{l,k}}$$

### 3.4. Overall Algorithm for Initial Parameter Construction

Based on the detailed derivation of the reference intervals, representative points, and initial belief degrees described above, this section presents the construction process of the initial parameters for the DD-IBRB model. The initial parameters determined by this algorithm will serve as the input to the inference mechanism of the DD-IBRB model and will be used for subsequent PV power prediction tasks. The complete execution steps are shown in Algorithm 1.
**Algorithm 1.** Construction of initial parameters**Input**: training dataset, clustering parameters K,δ,ς, maximum iteration, density parameter γ, result reference values **Output**: reference intervals $I_{j}$, representative points within reference intervals $I_{k}^{R}$, initial belief degrees $\beta_{l,k}$ **Procedure:** 1: Load training dataset 2: Extract dataset, antecedent attributes values and result values 3: ▷ FCM-based reference interval partition 4: for each feature f = 1, …, F do 5.  for K = $K_{\min},\ldots K_{\max}$ do 6:     Run FCM on dataset and compute BIC 7:  end for 8:  Select optimal K with minimum BIC 9:  Generate reference intervals 10: end for 11: ▷ DWDS key point selection  12: for each interval $\rho_{i}$ of feature do 13:   Compute representative density ρ and distance to center 14:   Select point with maximum score $S_{i}=\frac{\rho_{i}}{1+V_{i}}$ 15: end for 16: ▷ Belief degree calculation using GIBM 17: for each training sample do 18:   Compute output membership based on result reference values 19:   Update rule belief degrees using Gaussian activation 20: end for 21: Normalize belief matrix $\beta_{l,k}$ 22: Extract reference intervals $\to I_{j}$ 23: Extract representative points $\to I_{k}^{R}$ 24: return $I_{j}$, $I_{k}^{R}$, $\beta_{l,k}$

## 4. Inference and Optimization Process of the DD-IBRB Model

Section 4.1 describes the inference process of the DD-IBRB model. Section 4.2 describes the optimization process of the DD-IBRB model.

### 4.1. Inference Process of the DD-IBRB Model

The DD-IBRB model improves rule matching and activation weight calculation. This overcomes the limitations of IBRB models, which treat different samples equally within the same reference interval. The model employs Gaussian functions to compute the matching degree, allowing the rule activation weights to be dynamically adjusted according to the input samples, thereby accurately reflecting the data characteristics. During inference, ER rules are used, treating each rule as a piece of evidence. By quantifying the belief degree, weight, and reliability of each rule, multiple pieces of evidence are effectively fused to produce reliable evaluation results. The specific steps are as follows:

Step 1: Interval matching degree calculation. In the DD-IBRB model, the matching degree between the input sample and the belief rules is used to evaluate the applicability of each rule to the current data. When a sample $x_{i}$ falls within a reference interval $I_{j}$, the corresponding rule is immediately activated. The matching degree is calculated as follows:

(27) $$\gamma_{i}^{k}=\exp\left(-\frac{\left(x_{i}-I_{j}^{R}\right)}{2\sigma_{j}^{2}}\right)$$

Step 2: Activated Rule Weight Calculation. On the basis of the matching degree, the activation weight of the rule for sample $x_{i}$ is calculated as follows:

(28) $$\theta_{i}^{k}=\psi +(1-\psi )\times \gamma_{i}^{k}\times w_{k}$$

where ψ∈[0,1] is the minimum activation weight preset by the experts, set to 0.5, and where $w_{k}$ is the weight of the rule.

Step 3: ER rule inference. The rules in the DD-IBRB modeling process are treated as evidence in the ER rules. Let the L pieces of independent evidence be denoted as $e_{i}(i=1,\ldots ,L)$, and the frame of discernment be denoted as $\Theta =\{D_{1},\ldots ,D_{N}\}$, representing all possible evaluation grades. Each piece of evidence $e_{i}$ can be represented by the following belief distribution:

(29) $$e_{i}=\{(D_{n},\beta_{n,i}),\;n=1,\ldots ,N;\;(\Theta ,\beta_{\Theta ,i})\},\;\;\;0\leq \beta_{n,i}\leq 1,\;\;\;\sum_{n=1}^{N}\beta_{n,i}\leq 1$$

where $\beta_{n,i}$ represents the belief degree that the evaluation scenario is assigned to grade $D_{n}$ under evidence $e_{i}$.

Assuming that the evidence weight is $w_{i}\in [0,1](i=1,\ldots ,L)$ and the evidence reliability is $r_{i}\in [0,1](i=1,\ldots ,L)$, the belief distribution incorporating both weight and reliability can be expressed as:

(30) $$m_{i}=\{(D_{n},{\tilde{m}}_{n,i}),\;\forall D_{n}\subseteq \Theta ;\;(\beta (\Theta ),{\tilde{m}}_{\beta (\Theta ),i})\}$$

where β(Θ) denotes the power set of Θ. Then, the combined probability mass ${\tilde{m}}_{n,i}$ is calculated as:

(31) $${\tilde{m}}_{n,i}=\left\{\begin{array}{ll} 0, & D_{n}=\emptyset \\ c_{rw,i}m_{n,i}, & D_{n}\subseteq \Theta ,\;D_{n}\neq \emptyset \\ c_{rw,i}(1-r_{i}), & D_{n}=\beta (\Theta ) \end{array}\right.$$

(32) $$m_{n,i}=w_{i}\beta_{n,i}$$

where the normalization coefficient is denoted by $c_{rw,i}=1/(1+w_{i}-r_{i})$, satisfying $\sum_{n=1}^{N}{\tilde{m}}_{n,i}+{\tilde{m}}_{\beta (\Theta ),i}=1$. The combined belief degrees of any two pieces of evidence are calculated as follows:

(33) $${\hat{m}}_{n,e(2)}=[(1-r_{i})m_{n,j}+(1-r_{j})m_{n,i}]+\underset{A\cap B=D_{n}}{\sum }\underset{A,B\subseteq \Theta }{\sum }m_{A,i}m_{B,j}$$

(34) $$\beta_{n,e(2)}=\left\{\begin{array}{ll} 0, & D_{n}=\emptyset \\ \frac{{\hat{m}}_{n,e(2)}}{\sum_{A\subseteq \Theta }{\hat{m}}_{A,e(2)}}, & D_{n}\subseteq \Theta ,\;D_{n}\neq \emptyset \end{array}\right.$$

Then, the joint belief degrees of L independent pieces of evidence $\beta_{n,e(L)}$ can be generalized as follows:

(35) $$\forall D_{n}\in \Theta ,\;{\hat{m}}_{n,e(k)}=[(1-r_{k})m_{n,e(k-1)}+m_{\beta (\Theta ),e(k-1)}m_{n,k}]+\underset{A\cap B=D_{n}}{\sum }m_{A,e(k-1)}m_{B,k}$$

(36) $${\hat{m}}_{\beta (\Theta ),e(k)}=(1-r_{k})m_{\beta (\Theta ),e(k-1)}$$

(37) $$m_{n,e(k)}=\left\{\begin{array}{ll} 0, & D_{n}=\emptyset \\ \frac{{\hat{m}}_{n,e(k)}}{\sum_{A\subseteq \Theta }{\hat{m}}_{A,e(k)}+{\hat{m}}_{\beta (\Theta ),e(k)}}, & D_{n}\neq \emptyset \end{array}\right.$$

(38) $$\beta_{n,e(k)}=\left\{\begin{array}{ll} 0, & D_{n}=\emptyset \\ \frac{{\hat{m}}_{n,e(k)}}{\sum_{A\subseteq \Theta }{\hat{m}}_{A,e(k)}}, & D_{n}\subseteq \Theta ,\;D_{n}\neq \emptyset \end{array}\right.$$

where k = 3, 4, …, L, and where $\beta_{n,e(k)}$ denotes the belief degree after fusing k pieces of evidence. Initialize $m_{n,e(1)}=m_{n,1}$ and $m_{\beta (\Theta ),e(1)}=m_{\beta (\Theta ),1}$. Through the above inference process, the aggregated evaluation result is calculated as follows:

(39) $$e(L)=\{(D_{n},\beta_{n,e(L)}),n=1,\ldots ,N;(\Theta ,\beta_{\Theta ,e(L)})\}$$

The final expected utility is as follows:

(40) $$y=\overset{N}{\underset{n=1}{\sum }}u(D_{n})\beta_{n,e(L)}+u(\Theta )\beta_{n,e(L)}$$

where $u(D_{n})$ is the utility value of grade $D_{n}$.

### 4.2. Optimization Process of the DD-IBRB Model

In the DD-IBRB model, the initial parameters are usually derived from historical observation data. However, such data are affected by factors such as weather fluctuations and sensor biases, introducing uncertainty and making it difficult to accurately represent the real behavior of the PV conversion system. For example, sudden changes in solar irradiance may cause drastic fluctuations in power output, leading to errors in the parameter system established solely based on historical data.

To reduce the impact of uncertainty on model parameters and make them better reflect the actual operating state, this study introduces the animated oat optimization (AOO) algorithm [37]. This algorithm has high convergence efficiency and optimization accuracy, making it suitable for parameter optimization in PV systems. However, when dealing with high-dimensional parameter optimization problems, the optimization results of AOO may be unstable. The optimization process may also cause the model parameters to lose their physical meaning. To overcome these issues, this paper proposes an improved MEAOO algorithm. In this algorithm, the initial population is divided into two groups: a development population and an exploration population. The former performs fine searches in the neighborhood of the optimal solution to improve the local exploitation ability, whereas the latter performs random searches across the global range to maintain population diversity and avoid falling into local optima. The two groups work collaboratively to achieve a balance between global and local searches. The optimization model uses the mean squared error (MSE) between the predicted and measured values as the performance metric. The objective function is constructed as follows:

(41) $$MSE(\beta ,r,w)=\frac{1}{WT}\overset{WT}{\underset{j=1}{\sum }}{\left(Value_{predict}-Value_{actual}\right)}^{2}$$

where WT represents the amount of test data. The optimization objective is as follows:

(42) minMSE(β,r,w)

However, the parameters of the DD-IBRB model after optimization may lose their physical significance. For example, a rule with an initial belief distribution of {0.1, 0.8, 0.1} may change to {0.7, 0.2, 0.1} after optimization. To ensure the rationality of key model parameters, constraint conditions are introduced into the DD-IBRB parameter optimization process, as shown below:

(43) $$\begin{array}{l} w_{\min}\leq w_{k}\leq 1\;\;\;(k=1,2,\ldots ,L),\;r_{\min}\leq r_{k}\leq 1\;(k=1,2,\ldots ,L)\\ {\left(\beta_{i,k}\right)}_{\min}\leq \beta_{i,k}\leq {\left(\beta_{i,k}\right)}_{\max}\;(i=1,2,\ldots ,N),{\;\sum }_{l=1}^{N}\beta_{l,k}=1\;(k=1,2,\ldots ,L) \end{array}$$

**Figure 3:**
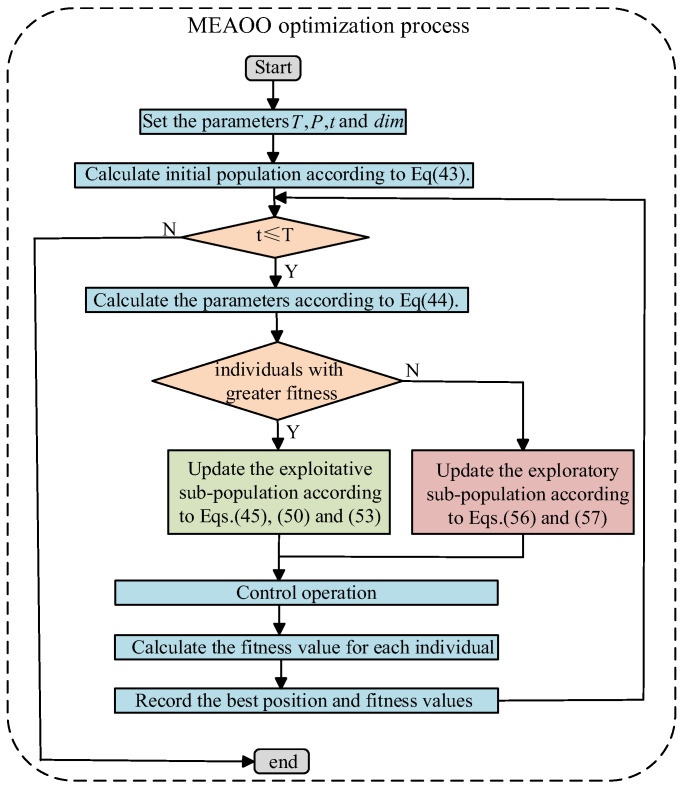
Flowchart of the MEAOO.

The main workflow of the MEAOO algorithm is shown in Figure 3. The specific optimization process is as follows:

Step 1 (Initialization and fitness calculation): Set the population size to P, the maximum number of iterations to T, and the search space to a dimension of dim. A seed-guided initialization is used to generate the initial population. From Formula (43), the population is constructed around the initial seed solution. This design accelerates entry into the effective search region. Each individual corresponds to a set of parameters to be optimized for the DD-IBRB model, and its fitness value is computed.

(44) $$\left\{\begin{array}{ll} \Omega_{i,:}=\Omega +\alpha \cdot (2\cdot q_{i}-1)\cdot (ub-lb) & ub,lb\;are\;consistent\\ \Omega_{i,j}=\Omega_{j}+\alpha \cdot (2\cdot q_{i,j}-1)\cdot (ub_{j}-lb_{j}) & ub,lb\;are\;inconsistent \end{array}\right.$$

where α is the disturbance coefficient, and where ub,lb represents the upper and lower bounds of the parameter constraints.

Step 2 (Parameter settings): The seed mass m, main awn length l, eccentric rotation coefficient ξ, and dynamic adjustment factor κ are computed via the following formulas:

(45) $$\left\{\begin{array}{l} m=0.5\times q^{\dim}\\ l=P\times q^{\dim}\\ \xi =0.5\times q^{\dim}\\ \kappa =1-{\left(\frac{t}{T}\right)}^{3} \end{array}\right.$$

where q is a random number within the interval [0, 1], and t denotes the current iteration number.

**Figure 4:**
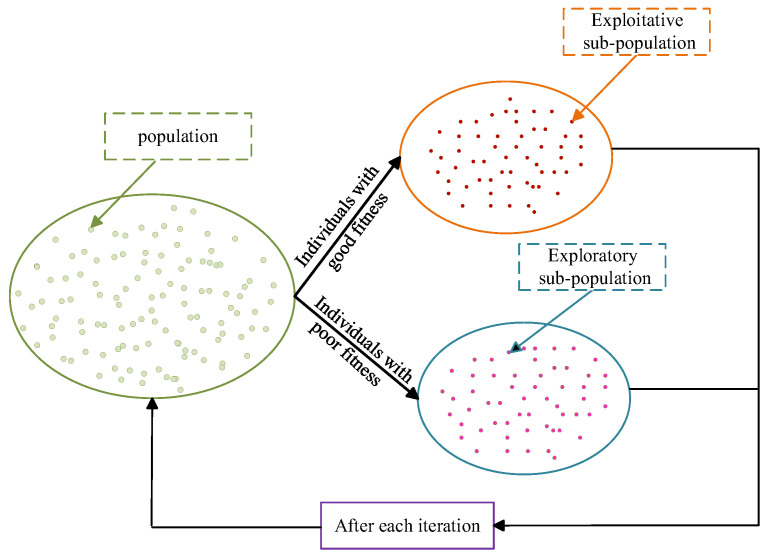
Multi-population division.

Step 3 (Subpopulation Division): As shown in Figure 4, the population is divided into two subpopulations according to the fitness ranking of individuals:

Development population: This population is composed of individuals with greater fitness, who are responsible for performing global exploration strategies.

Exploration population: This population is composed of individuals with lower fitness, who are responsible for performing local exploitation strategies.

Step 4 (Update the exploitative sub-population): For each individual $\Omega_{i}$ in the development population, three strategies are used to update its position.

Step 4.1. Obstacle-free (hygroscopic rolling): A local fine search operation that simulates the rolling behavior caused by the hygroscopic twisting of the main awn. The hygroscopic rolling method in MEAOO is expressed as Formula (45).

(46) $$\Omega_{i}(t)=\Omega_{best}+G+\kappa \times Levy(\dim)⊗\Omega_{best}$$

where the position vector of the current best solution is denoted as $\Omega_{best}$, which is updated at each iteration. The coefficient vector is denoted as G, and it can be calculated via Equations (46) and (47). Levy(dim) represents a random step-length vector generated based on the Lévy distribution with a dimension of dim, which can be obtained via Formulas (48) and (49).

(47) $$G=(m\times \xi +l^{2})\times q_{\dim}{\left(-X,X\right)}_{\dim}$$

(48) $$X=ub-\left|ub\times \frac{t\times \sin(2\times \pi \times q)}{T}\right|$$

where A is the amplitude parameter, and where $q_{\dim}(-X,X)$ is a random matrix within the range $\left[-X,X\right]$.

(49) $$Levy(\dim)=0.01\times \frac{q_{1}\times \sigma_{1}}{{\left|q_{2}\right|}^{1/\sigma_{2}}}$$

(50) $$\sigma ={\left(\frac{\Gamma (1+\sigma_{2})\times \sin\left(\frac{\pi \times \sigma_{2}}{2}\right)}{\Gamma \left(\frac{1+\sigma_{2}}{2}\right)\times \sigma_{2}\times 2^{\left(\frac{\sigma_{2}-1}{2}\right)}}\right)}^{1/\beta }$$

where $q_{1}$ represents the randomness of the step’s direction and magnitude, $q_{2}$ denotes the current velocity vector, $\sigma_{1}$ is the scale parameter of the Lévy distribution controlling the width and range of the step length distribution, and $\sigma_{2}$ is the Lévy distribution’s exponent parameter that governs the shape and heavy-tail characteristics of the step length distribution. Γ denotes the Gamma function.

Step 4.2. Energy ejection (with obstacles). When a seed encounters an obstacle, it stores energy and performs a parabolic ejection motion. The mathematical process of this behavior can be described as follows:

(51) $$\Omega_{i}(t)=\Omega_{best}+H+\kappa \times Levy(\dim)⊗\Omega_{best}$$

(52) $$H=\frac{2\times \vartheta \times {\left(\Delta l\right)}^{2}\times \sin(2∡)}{mg}\times q_{\dim}{\left(-Y,Y\right)}_{\dim}\times (1-\iota )$$

(53) $$\left\{\begin{array}{l} Y=ub-\left|ub\times \frac{t\times \cos(2\times \pi \times q)}{T}\right|\\ \vartheta =0.5+0.5\times q\\ \Delta l=3\times \frac{r}{\dim}\\ ∡=\pi \times q\\ \iota =\frac{1}{\pi }\cdot e^{\frac{q^{\prime }}{T}} \end{array}\right.$$

where g is the gravitational acceleration, H is the ejection vector, Y is a dynamically adjusted range coefficient used to constrain the search space boundaries of the ejection behavior, $q^{\prime }$ is a random number within the range $\left[0,T\right]$, ϑ is the elasticity coefficient, Δl represents the variation in the main awn length, ∡ is the ejection angle, and ι denotes the air resistance coefficient.

Step 4.3. Quadratic interpolation. A quadratic function is constructed to approximate the local extremum, and a new candidate solution is generated via the current best individual $\Omega_{best}(\Omega_{best,1},\ldots ,\Omega_{best,\dim})$ and two random individuals $\Omega_{1}(\Omega_{1,1},\ldots ,\Omega_{1,\dim})$ and $\Omega_{2}(\Omega_{2,1},\ldots ,\Omega_{2,\dim})$. The new solution is generated via the following Formula (53):

(54) $$\Omega_{i}(t)=\frac{(\Omega_{1,i}^{2}-\Omega_{2,i}^{2})\cdot v_{best}+(\Omega_{2,i}^{2}-\Omega_{best,i}^{2})\cdot v_{1}+(\Omega_{best,i}^{2}-\Omega_{1,i}^{2})\cdot v_{2}}{2((\Omega_{1,i}-\Omega_{2,i})\cdot v_{best}+(\Omega_{2,i}-\Omega_{best,i})\cdot v_{1}+(\Omega_{best,i}-\Omega_{1,i})\cdot v_{2})}$$

where i=1,…,dim. $v_{best},v_{1},v_{2}$ represent the fitness values corresponding to each individual.

If the denominator of a certain dimension equals zero, the value of that dimension is randomly selected from the current best individual $\Omega_{best}$:

(55) $$\Omega_{:,j}=\Omega_{best,i}$$

where j denotes the dimension with a zero denominator, and i represents the dimension randomly selected from $\Omega_{best}$.

Step 5 (Update exploratory sub-population): For each individual $\Omega_{i}$ in the exploration subpopulation, two strategies are used to update its position:

Step 5.1. Natural element dissemination. Seeds are dispersed randomly by natural forces such as wind, water, or animals, generating a random disturbance Z. The position of each individual is then updated according to its index as follows:

(56) $$Z=\kappa \cdot \frac{\pi \times (2\times q_{\dim}-1)⊗ub}{\pi }$$

(57) $$\left\{\begin{array}{ll} \Omega_{i}(t+1)=\frac{1}{P}\sum_{p=1}^{P}\Omega_{i}(t)+Z, & if\;\mathrm{mod}\;(i,P/10)=0\\ \Omega_{i}(t+1)=\Omega_{best}+Z, & if\;\mathrm{mod}\;(i,P/10)=1\\ \Omega_{i}(t+1)=\Omega_{i}(t)+Z, & else \end{array}\right.$$

where $\Omega_{i}(t)$ represents the ith individual in the population at iteration t, and where $\Omega_{i}(t+1)$ represents the ith individual at iteration t+1.

Step 5.2. Random seed expansion. Seeds expand their search range by increasing the absolute value of their position vectors.

(58) $$\Omega_{i}(t+1)=\Omega_{i}(t)\times (1+q)$$

Step 6: Control operation. During the sampling process, a control procedure is used to ensure that all individuals satisfy the parameter constraints.

Step 7: After each iteration, the fitness of all individuals is recalculated, and the population is re-divided according to the current fitness values.

## 5. Case Study

Section 5.1 describes the dataset used in this study, Section 5.2 outlines the structure of the DD-IBRB model for PV power prediction, Section 5.3 analyzes the case study results, Section 5.4 evaluates the performance of the optimization algorithm, Section 5.5 presents the model complexity analysis, Section 5.6 conducts comparative experiments to verify the advantages of the model, and Section 5.7 analyzes the generalization ability using another PV dataset.

### 5.1. Dataset Description

**Figure 5:**
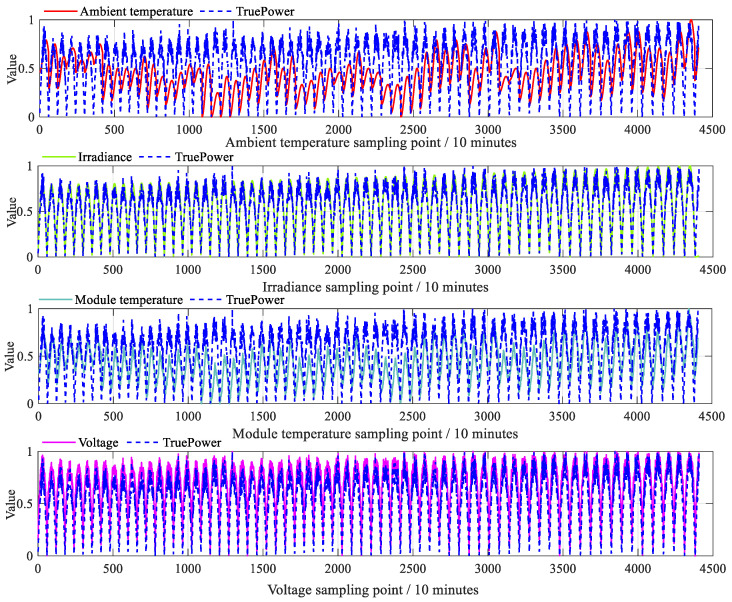
Distribution of data for the PV.

To verify the effectiveness of the proposed model, this study employs a PV power prediction dataset as a case study. The PV power data were obtained from https://aistudio.baidu.com/datasetdetail/147402 (accessed on 3 January 2026). The sampling period ranges from 1 January 2018, to 11 March 2018, with a sampling interval of 10 min. The dataset covers the time span from winter to early spring. The dataset has been desensitized. Since PV modules generate electricity only in the presence of solar irradiance, both irradiance and PV power are zero in the dataset from approximately 17:00 to 07:10 the following day. To ensure effective model training, these nighttime zero-power samples were removed. Only samples collected under solar irradiance conditions were retained. After screening, 4407 samples remained for model training and testing, of which 2938 were used for training and 1469 for testing. All data were normalized before use. As shown in Figure 5, attributes including ambient temperature, solar irradiance, module temperature, and voltage all influence the output power.

### 5.2. Construction of the DD-IBRB Model for PV Power Generation Prediction

To address the modeling problem caused by insufficient expert knowledge, the DD-IBRB model is constructed in this section. To effectively determine the number of reference intervals and reduce model complexity, the number of cluster centers is limited to no more than 15 based on the data distribution. The FCM-BIC method is used to obtain the cluster centers for each clustering number and to evaluate the clustering results. A set of cluster center values is then generated, and the midpoints between adjacent centers are taken as the boundaries of the reference intervals. The reference intervals of the antecedent attributes are shown in Table 2.

**Figure 6:**
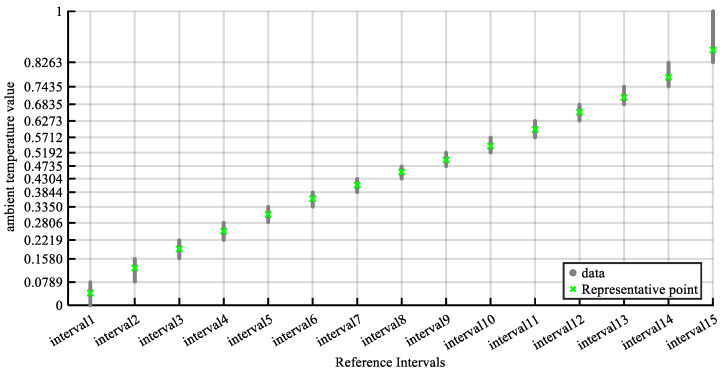
Representative points for the ambient temperature within the reference interval.

**Figure 7:**
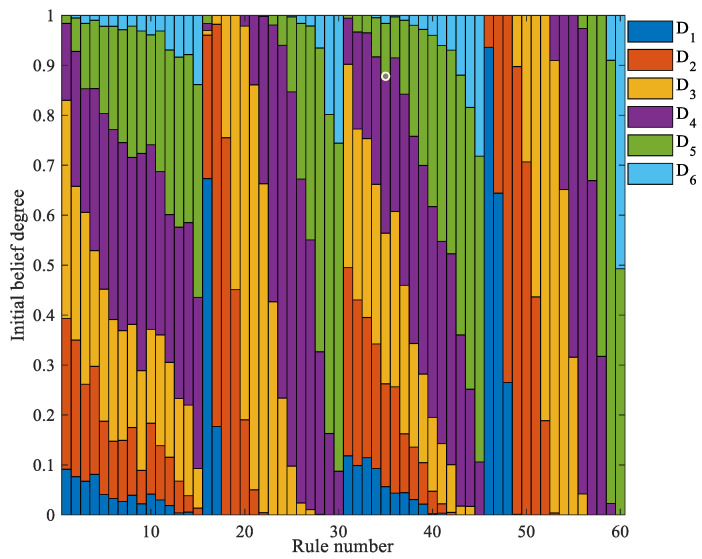
Initial belief distribution.

After the reference intervals are determined, the DWDS algorithm is applied to identify the representative points of each interval. As shown in Figure 6, the green “×” symbols denote the representative points within each interval, whereas the gray dots represent other sample points. For example, the representative point of Interval 5 [0.2806, 0.3350] is 0.3093. The GIBM strategy is subsequently used to calculate the initial belief degrees of each rule, as shown in Figure 7.

Through the above three steps, a portion of the initial parameters required by the model can be obtained. In this experiment, the resulting reference values are provided by experts, as shown in Table 3. Both the initial rule weights and rule reliabilities are set to 1 and used as part of the model’s initial parameters. After parameter initialization, the initial DD-IBRB model for PV power generation prediction is established. The initial model specifically includes the initial belief degrees, rule reliabilities, and rule weights, as shown in Table 4.

The belief degrees, rule weights, and rule reliabilities are further optimized via the MEAOO algorithm. During this process, constraints are imposed on these parameters to preserve their physical meaning and ensure reliability in practical applications. In the DD-IBRB model used for PV power prediction, the belief rules are described as follows:

(59) $$\begin{array}{l} R_{k}:If\;ambient\;temperature\in [a_{1},b_{1}]\vee irradiance\in [a_{2},b_{2}]\vee \\ \;\;\;\;\mathrm{mod}ule\;temperature\in [a_{3},b_{3}]\vee voltage\in [a_{4},b_{4}]\\ \;\;\;\;\;\;Then\;PV\;value\;is\left\{\begin{array}{l} \left(D_{1},\beta_{1,k}\right),\left(D_{2},\beta_{2,k}\right),\left(D_{3},\beta_{3,k}\right)\\ \left(D_{4},\beta_{4,k}\right),\left(D_{5},\beta_{5,k}\right),\left(D_{6},\beta_{6,k}\right) \end{array}\right\}\left(\sum_{n=1}^{6}\beta_{n,k}=1\right)\\ \;\;\;with\;rule\;weight\;w_{k},\;rule\;reliability\;r_{k}\left(k=1,\ldots ,L\right)\\ \;\;\;and\;the\;representative\;po\mathrm{int}\;I_{k}^{R} \end{array}$$

### 5.3. Experimental Results Analysis

During the model optimization stage, the maximum number of iterations for the MEAOO algorithm is set to 300, the initial population size is set to 180, and the parameter constraint condition is $0.5\leq w_{k}\leq 1,\;0.5\leq r_{k}\leq 1\;$, $\beta_{i,k}-0.15\leq \beta_{i,k}\leq \beta_{i,k}+0.15,\sum_{l=1}^{N}\beta_{l,k}=1\;$. This algorithm is used to optimize the initial parameters of the DD-IBRB model. The detailed results of the optimized parameters are shown in Table 5.

To comprehensively evaluate the performance of the DD-IBRB model, several error metrics are selected. The mean squared error (MSE), mean absolute error (MAE), coefficient of determination ($R^{2}$), and symmetric mean absolute percentage error (SMAPE) are used to systematically measure the accuracy of the prediction results. In addition, the standard deviation (SD) of MSE is recorded to assess the stability of the model.

**Figure 8:**
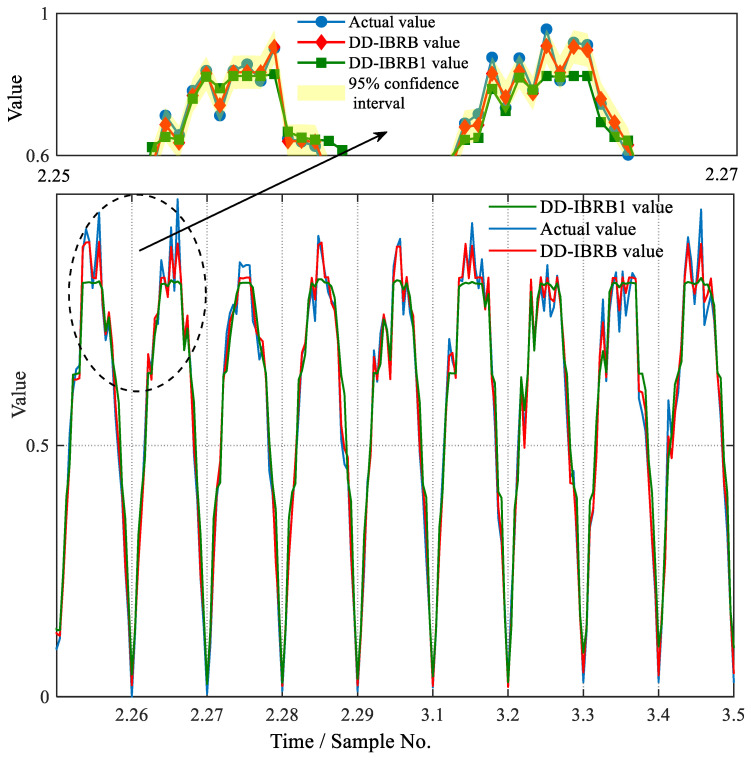
Prediction results of the DD-IBRB1 model and DD-IBRB model.

In this study, the comparison between the predicted and actual values is shown in Figure 8. To enable clearer visual analysis, we selected prediction results from 25 February to 5 March for plotting. This period captures the winter-to-spring transition, making the data variation characteristics more representative. The 95% confidence interval of DD-IBRB in Figure 8 effectively envelops the true values, demonstrating its superior uncertainty quantification reliability and accuracy during power fluctuations. And it can be seen from the figure that the DD-IBRB model predicts photovoltaic power generation more accurately than DD-IBRB1. DD-IBRB1 is the initial model without optimization. Its prediction curve generally follows the variation in the actual values. As can be seen from Table 6, DD-IBRB1 has an MSE of 0.00270. This indicates that the initial parameters generated directly from data already provide high modeling accuracy. However, near power fluctuation points, DD-IBRB1 shows noticeable deviations and fails to capture sudden changes accurately. This limitation is clearly illustrated in the subplots of Figure 8. The DD-IBRB model is an improved version of DD-IBRB1, obtained through parameter optimization. Its prediction curve almost completely coincides with the actual values. The MSE decreases to 0.00056 and MAE to 0.019, as shown in Table 6. The error levels are greatly reduced, significantly improving prediction accuracy in mutation regions. To further analyze the model performance, the DD-IBRB model is compared with the basic IBRB [38] and IBRB0 models. The reference interval of IBRB is evenly divided based on the range of each attribute. The initial confidence level is randomly generated within [0, 1]. IBRB0 is the IBRB model without any optimization algorithm. As can be seen from Table 6, in terms of both model structure and optimization algorithm, the model performance can be further improved. These results demonstrate that the model has strong predictive ability in practical applications and can accurately reflect variations in PV power generation.

To further verify the robustness of the optimized DD-IBRB model, multiple sets of experiments were designed in this study. Four different training data proportions were selected. For each proportion, ten independent experiments were conducted to reduce the influence of random factors. The average performance of the model was evaluated using the mean of the experimental results. The standard deviation of the MSE was used to measure model stability. The detailed results are presented in Table 7. The experimental results show that the MSE continuously decreases as the proportion of training data increases. Meanwhile, under all data proportions, the standard deviations of the MSE across the ten experiments remain small. This indicates that the DD-IBRB model exhibits good robustness under different data configuration conditions. It can also stably demonstrate its modeling capability when sufficient training data are provided.

### 5.4. Performance Analysis of the Optimization Method

**Figure 9:**
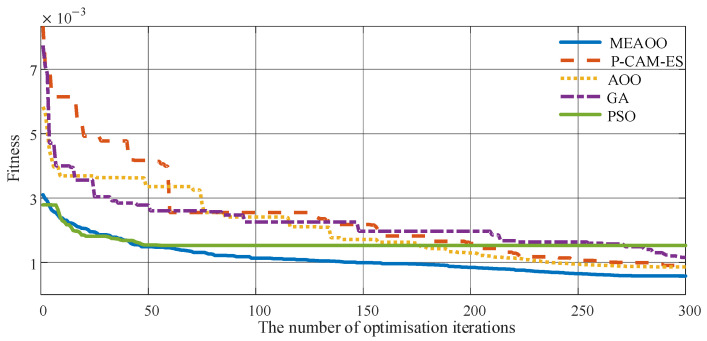
Decline in fitness of the predictive model via five optimization algorithms.

**Figure 10:**
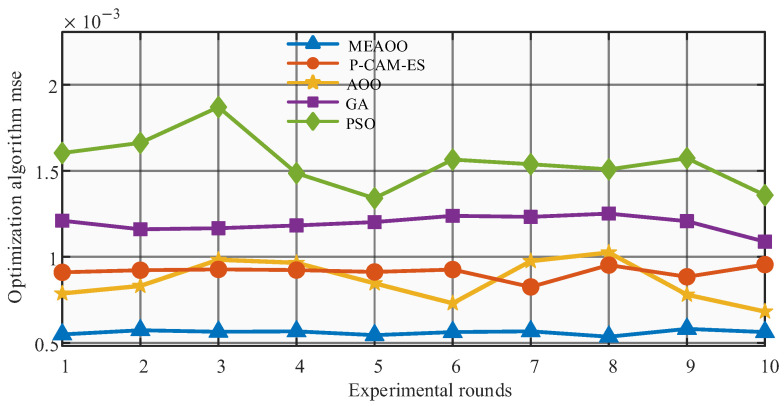
MSE of optimization algorithm.

The variation in fitness values during the training process for five optimization algorithms is presented in Figure 9. The MSE results of ten experiments for different algorithms are shown in Figure 10. From Figure 9, it can be seen that MEAOO achieves the fastest fitness decline and reaches the minimum value within few iterations, demonstrating excellent global optimization ability and convergence performance. In contrast, particle swarm optimization (PSO) decreases quickly at the beginning but easily falls into local optima, resulting in a final fitness higher than MEAOO. Projected covariance matrix adaptation evolution strategy (P-CAM-ES) shows a slower decline initially, but its performance gradually improves later. Both AOO and GA decline rapidly at the beginning and flatten in the middle and later stages, with AOO ultimately outperforming genetic algorithm (GA).

Figure 10 further verifies the stability and average performance of different optimization algorithms across multiple experiments. MEAOO consistently achieves the lowest MSE with the smallest fluctuations, demonstrating the best convergence accuracy and stability. PSO shows relatively high MSE with large fluctuations, while P-CAM-ES, GA, and AOO exhibit moderate stability. Table 8 further shows that MEAOO obtains the lowest MSE and RMSE, as well as the smallest standard deviation, indicating its clear advantage in optimization accuracy and result consistency. In summary, MEAOO outperforms the other algorithms in fitness decline speed, final convergence accuracy, and stability across multiple experiments.

**Figure 11:**
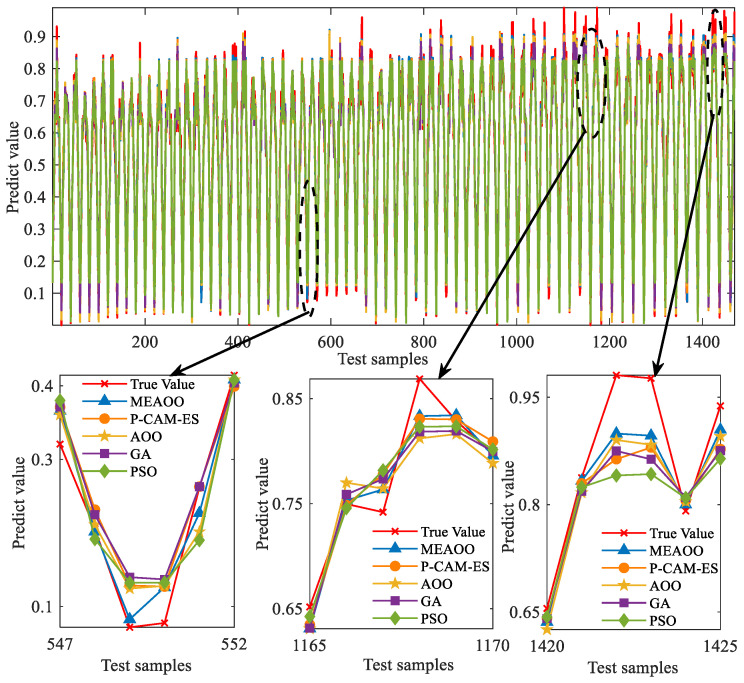
Predictive model fit via five optimization algorithms.

Figure 11 shows the comparison between the predicted values and the actual values for each optimization algorithm. Overall, all algorithms capture the data trends well, but noticeable differences appear at local peaks. The magnified view in Figure 11 reveals that MEAOO’s predictions most closely match the actual values. This shows that MEAOO is more stable and robust when confronted with sudden fluctuations or highly volatile regions.

**Figure 12:**
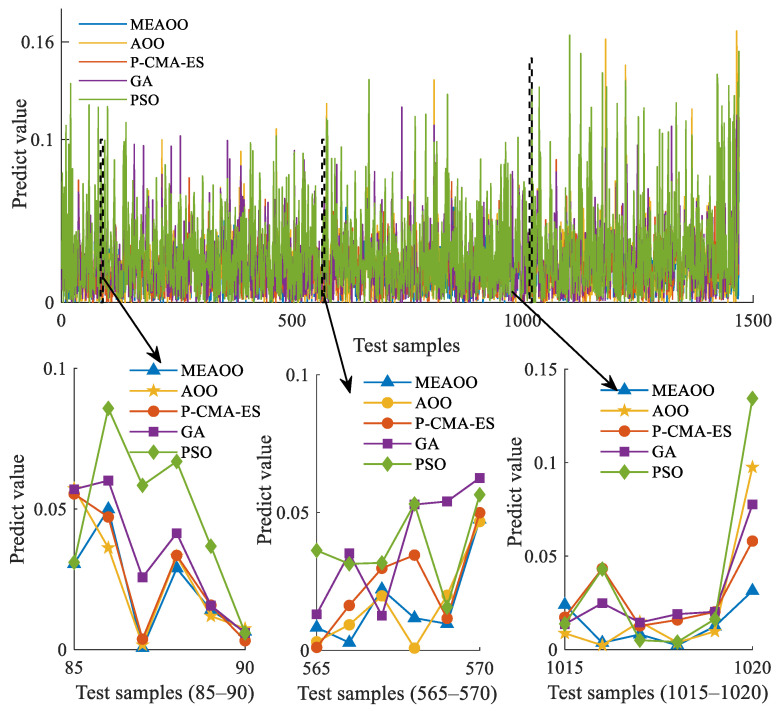
Error diagram for five optimization algorithms.

Figure 12 illustrates the error comparison among the five optimization algorithms on the test set, where the error value is defined as:

(60) $$error(x_{i})=\sqrt{\left|predict(x_{i})\right|-actual(x_{i})},(i=1,2,\ldots ,140)$$

where $predict(x_{i})$ is the model’s predicted value for the ith sample, $actual(x_{i})$ is the true value of the ith sample, and $error(x_{i})$ represents the resulting error. Figure 12 shows that the model via the MEAOO algorithm has the smallest deviation at most sample points, demonstrating the best stability and accuracy. In contrast, PSO exhibits large error fluctuations. GA shows noticeable error variations but performs better than PSO. AOO has moderate error changes, with only a few points slightly higher. P-CMA-ES has generally small errors, though a few points still fluctuate. Overall, MEAOO demonstrates superior fitting ability and generalization stability.

In terms of stability, AOO and P-CMA-ES show large fluctuations in prediction errors across different sample points. This indicates that their prediction process is not stable and performance may decrease when handling out-of-distribution or new samples. In contrast, the MEAOO-based DD-IBRB prediction model demonstrates superior accuracy and stability.

### 5.5. Computational Complexity and Scalability Analysis

To evaluate the practicality of the model, this section analyzes its computational cost, space complexity, and scalability for large-scale applications.

(1) Parameter generation

Let the number of samples in the training set be W, the feature dimension M, the number of clusters K, the maximum number of iterations be T, and the number of result grades N. In the parameter generation process, the total complexity is O(W⋅K⋅M+K⋅M+N⋅M). As shown in Table 6, in the absence of expert knowledge, the model’s MSE is 0.02280. After adopting the parameter generation method proposed in this paper, the MSE is significantly reduced to 0.00270. The additional computational overhead remains within an acceptable range.

(2) Multi rule activation and fusion

The activation rules of multiple attributes are combined, and the output belief degrees are calculated using the ER rules. Each attribute input activates at most two rules, resulting in $2^{T}$ rule combinations for a single sample. For a dataset containing M attributes and N result grades, the computational complexity of a single ER inference is O(W⋅N), and the total computational complexity is $W\cdot N\cdot 2^{T}$. The complexity increases exponentially as the number of features in the dataset grows. However, feature selection techniques, such as the extreme gradient boosting algorithm [39], can be employed to evaluate the relative importance of meteorological variables and reduce feature redundancy. In practical PV prediction scenarios, many meteorological variables exhibit strong correlations. By retaining the most informative features while eliminating redundant ones, the effective dimensionality of the input space can be controlled, thereby mitigating the rule explosion problem caused by high-dimensional inputs. Alternatively, a hierarchical DD-IBRB structure can be considered, in which features are grouped to construct multiple BRBs. This approach effectively alleviates the challenges caused by high-dimensional features. Therefore, the model exhibits excellent scalability in the reasoning phase after deployment and can efficiently handle massive prediction requests.

(3) Optimization algorithm

The complexity of the MEAOO optimization algorithm primarily depends on the population size P, the parameter dimension dim. The overall complexity is P⋅dim.

(4) Overall computational burden.

Although the DD-IBRB model requires more resources than the IBRB model, its operations are highly parallelizable, which can significantly accelerate the iterative optimization process.

Note: The experiments were conducted on a system with an Intel(R) Core(TM) i9-14900HX processor (2.20 GHz), 16 GB of RAM, and Windows 11 Professional, implemented in the MATLAB 2024b environment. The maximum number of clusters was set to 15, and the number of iterations was 300. After 10 repeated runs, the average running time was 150 s. The computational cost is mainly concentrated in the model parameter generation and optimization stages; once the model is constructed, the prediction time for a single run is only at the millisecond level. This indicates that the proposed algorithm can meet real-time response requirements in scenarios such as photovoltaic power prediction while ensuring prediction accuracy.

### 5.6. Comparative Experiment

To verify the effectiveness of the DD-IBRB model, comparative experiments are conducted in two parts. Each method is tested in 10 runs to ensure the stability and reliability of the results. To further verify the effectiveness of performance improvement, we conducted the Mann–Whitney U test between DD-IBRB and other models to evaluate statistical significance.

**Figure 13:**
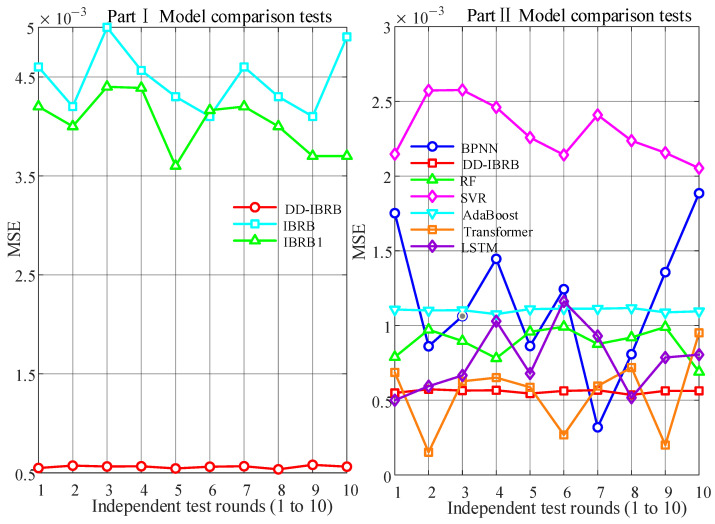
Comparison with other models using a 2:1 training-to-test ratio.

In the first part, the DD-IBRB model is compared with several IBRB models. These models include the basic IBRB and the IBRB model with rule matching degree calculation (IBRB1) [40]. Both IBRB1 and IBRB use the same method for reference interval division. The MEAOO algorithm is used to optimize key parameters to improve accuracy. As shown in Part I of Figure 13, with a 2:1 train–test split ratio, DD-IBRB achieves the lowest MSE across 10 runs, showing higher stability and precision. Table 9 further confirms this result. The MSE of DD-IBRB is only 0.00056, with the smallest standard deviation, indicating low fluctuation and strong robustness. The results of the statistical tests show that all *p*-values of the comparisons in the first part are less than 0.001, indicating that the improvement of DD-IBRB is statistically significant.

In the second part, the DD-IBRB model is compared with other advanced models. Several classical models are selected, including support vector regression (SVR), back-propagation neural networks (BPNN), random forests (RF), AdaBoost, long short-term memory (LSTM), and Transformer. The parameter settings of these models are provided in Appendix A Table A2. The experiments are analyzed from three aspects:

(1) Point prediction accuracy and stability: As shown in Part II of Figure 13 and Table 10, across different training-to-testing ratios, the DD-IBRB model consistently achieved strong predictive performance while demonstrating significantly superior prediction stability compared to baseline methods. In particular, at a 1:2 training-to-testing ratio, DD-IBRB obtained an MSE of 0.00130 with a standard deviation of only 0.00020, clearly outperforming Transformer and other baseline approaches in terms of stability. Overall, while the Transformer may yield slightly lower MSE in some cases, DD-IBRB exhibited better overall performance, especially regarding prediction stability and robustness. Statistical tests indicate that in most evaluation scenarios, DD-IBRB shows statistically significant improvements over classic baselines (SVR, BPNN, RF, AdaBoost), with *p*-values less than 0.01 in most cases. While the differences between DD-IBRB and LSTM or Transformer are not statistically significant for some metrics (*p* ≥ 0.01), DD-IBRB excels in prediction stability, making it more practical and reliable for real-world PV prediction.
(2) Interval prediction and uncertainty quantification: To further evaluate the models’ ability to quantify uncertainty, Table 11 presents the interval prediction performance of each model at a 2:1 ratio under a 95% confidence level. The results indicate that the baseline models often struggle to balance coverage and precision. For example, the coverage probabilities of the Transformer (87.01%) and LSTM (92.65%) do not reach the 95% level. In contrast, the DD-IBRB model achieves a PI coverage probability (PICP) of 95.37% while maintaining a relatively narrow PI normalized average width (PINAW) of 0.0928, exceeding the preset 95% confidence level. This demonstrates that DD-IBRB not only provides accurate point predictions but also achieves an optimal balance between reliability and precision in the generated confidence intervals.
(3) Interpretability: The DD-IBRB model provides a transparent reasoning mechanism. Although BPNN, Transformer and LSTM sometimes produce slightly better results, their structures are not interpretable. This indicates that DD-IBRB has better applicability in engineering practice.

**Table 10:** Comparison with other models under different training-to-test ratios.

Ratio	Model	MSE	SD (MSE)	MAE	$R^{2}$	SMAPE	*p*-Value
1:2	DD-IBRB	0.00130	0.00020	0.022	0.97	6.28%	/
	BPNN	0.00199	0.00112	0.031	0.96	10.78%	0.00317
	SVR	0.00608	0.00074	0.054	0.90	15.82%	<0.001
	RF	0.00236	0.00028	0.029	0.96	9.24%	<0.001
	AdaBoost	0.00163	0.00003	0.030	0.97	9.23%	<0.001
	Transformer	0.00144	0.00051	0.027	0.97	8.31%	0.06970
	LSTM	0.00158	0.00039	0.028	0.97	8.56%	0.04410
1:1	DD-IBRB	0.00083	0.00007	0.020	0.98	5.99%	/
	BPNN	0.00165	0.00089	0.028	0.97	9.17%	0.00136
	SVR	0.00317	0.00003	0.043	0.94	14.47%	<0.001
	RF	0.00130	0.00020	0.021	0.97	6.97%	<0.001
	AdaBoost	0.00125	0.00002	0.029	0.98	9.65%	<0.001
	Transformer	0.00093	0.00022	0.024	0.98	7.90%	0.03710
	LSTM	0.00098	0.00040	0.022	0.98	8.02%	0.03710
2:1	DD-IBRB	0.00056	0.00001	0.019	0.99	6.01%	/
	BPNN	0.00116	0.00047	0.025	0.98	8.68%	0.00141
	SVR	0.00230	0.00019	0.041	0.96	14.49%	<0.001
	RF	0.00088	0.00009	0.019	0.98	6.91%	<0.001
	AdaBoost	0.00110	0.00002	0.028	0.98	10.16%	<0.001
	Transformer	0.00054	0.00025	0.018	0.99	7.61%	0.07020
	LSTM	0.00076	0.00021	0.022	0.99	7.83%	0.01290

**Table 11:** Comparison of 95% prediction interval performance of various models under a 2:1 ratio.

Model	PINAW	PICP
DD-IBRB	0.0928	95.37%
BPNN	0.1117	91.75%
SVR	0.1276	79.19%
RF	0.1165	95.50%
AdaBoost	0.1294	98.97%
Transformer	0.0700	87.01%
LSTM	0.1096	92.65%

### 5.7. Generalization Capability Analysis

#### 5.7.1. Experimental Analysis

**Figure 14:**
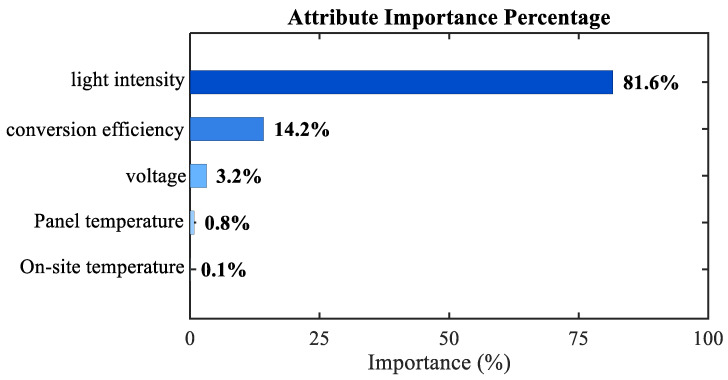
Attribute importance analysis.

Another PV power dataset sourced from https://aistudio.baidu.com/datasetdetail/104745 (accessed on 3 January 2026) was selected for analysis. The importance of the input features was evaluated via the extreme gradient boosting algorithm. Figure 14 shows that the importance of light intensity and conversion efficiency accounts for more than 90% of the total contribution. Therefore, irradiance and conversion efficiency were selected as the two input variables, and PV power output was set as the output variable. The experiment used 3402 samples for training and 1698 samples for testing.

**Figure 15:**
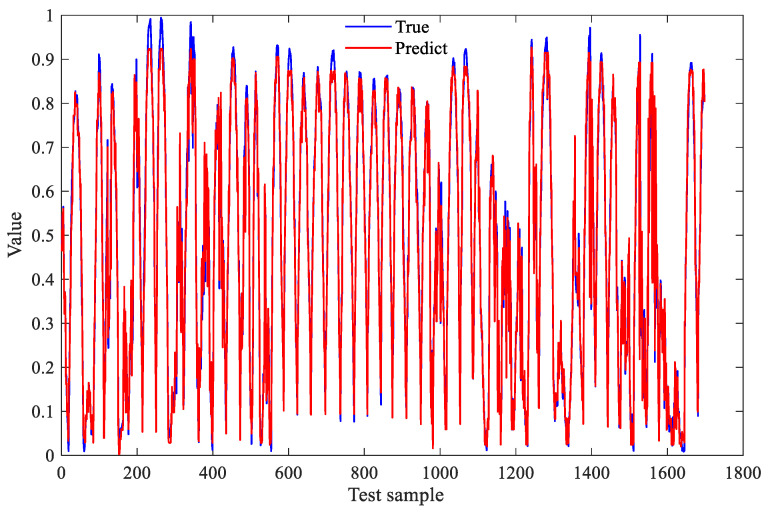
Prediction results of the DD-IBRB model.

The experiment set the maximum number of clusters to 15, following a procedure similar to Section that in 5.2. The MSE was adopted as the objective function. Most test samples were well predicted, and the fitting results are shown in Figure 15. As shown in Table 12, the evaluation metrics are an MSE of 0.00102 and an MAE of 0.0227. These results indicate that the model exhibits excellent prediction accuracy and low error levels, demonstrating strong generalizability and stability when handling unseen data, making it suitable for high-reliability prediction tasks in practical applications.

#### 5.7.2. Comparative Experimental Analysis

To evaluate the performance of the proposed method, two sets of comparative experiments are conducted for predictive analysis. Each method is tested in ten runs. In the first part, the DD-IBRB model is compared with the IBRB and DD-IBRB models via different optimization algorithms. In the second part, it is compared with other advanced models.

As shown in Table 12, the overall performance of the DD-IBRB model is significantly superior to that of the other models. This demonstrates that, even under conditions of limited expert knowledge, DD-IBRB can generate initial parameters from data and, through parameter-constrained optimization, maintain the physical interpretability of the model while improving prediction accuracy.

In this study, two PV power datasets are used to verify the DD-IBRB model. The experiments in Section 5 fully demonstrate the effectiveness and accuracy of the proposed model. The conclusions are summarized as follows:

(1) In the DD-IBRB model, experts only need to provide the result grades and the prior probability distributions. Then, by combining data-driven methods, the parameters required by the model can be accurately constructed. Compared with the IBRB and IBRB1 models, the DD-IBRB model achieves a lower MSE of 0.00056 and thus higher prediction accuracy.

(2) Compared with other advanced models, the DD-IBRB model not only shows good stability across multiple experiments but also provides a transparent reasoning engine. In contrast, the internal structures of other models are invisible.

## 6. Conclusions

In some engineering applications, the IBRB model faces difficulties due to limited expert knowledge during the modeling process. To address this problem, a data-driven IBRB (DD-IBRB) model is proposed. The DD-IBRB model maintains the strengths of the traditional IBRB framework while reducing the reliance on expert knowledge during modeling. It uses a data-driven approach to build reference intervals for antecedent attributes and belief degrees for consequents, improving its modeling capability. In addition, by selecting representative points within each reference interval, the model enables adaptive rule generation on the basis of data samples. Finally, a constrained optimization algorithm is employed to further improve prediction accuracy while maintaining model rationality. Experiments conducted on two photovoltaic power prediction datasets demonstrate the effectiveness and generalizability of the proposed DD-IBRB model.

Across multiple comparative experiments, the DD-IBRB model achieves superior results and exhibits greater stability than other machine learning models do. However, while the MEAOO algorithm used in the optimization process effectively improves model performance, its multi-population evolutionary mechanism may lead to higher computational costs. This presents a challenge that can be further explored in future research.

Another limitation of this study is the relatively limited size of the available PV datasets. Although the datasets used in this study are publicly available and have been employed in related studies, the limited data scale may still affect the comprehensive evaluation of the model’s generalization ability. In future work, longer-term PV datasets (e.g., year-long data covering different seasonal conditions) will be utilized to further validate the generalization performance of the proposed model.

## Figures and Tables

**Table 1:** Relevant studies and their problems.

Classification	Article	Mechanism	Limitation
Statistical models	[6,7,8,9]	Statistical models do not require a deep understanding of the internal mechanisms of the PV system, focusing only on the input and output.	Although statistical models can achieve high prediction accuracy, they require large amounts of data for model construction and often lack interpretability [10,11].
Physical models	[12,13,14]	Physical models describe the operating mechanisms of PV systems and can calculate their key design parameters.	The prediction accuracy of physical models depends on precise meteorological data and complete information about PV cells. In practice, parameters may be incomplete, and weather forecasts may be inaccurate. As a result, the modeling often cannot reach the desired level [15].
Hybrid approaches	[16]	In this paper, the focus is on the belief rule base (BRB) in hybrid approaches. In existing studies, the BRB model has strong nonlinear modeling capability, enabling it to effectively represent the detailed causal relationships between antecedent attributes and outcomes. This also makes it highly effective in handling uncertainty, providing decision-makers with more accurate and reliable prediction results. IBRB model has demonstrated significant advantages in handling multi-attribute decision-making problems, making it more suitable for practical engineering applications.	However, when initially establishing an IBRB model, expert knowledge in the relevant field is still needed to define the reference intervals and belief degrees. In some engineering problems, sufficient expert knowledge may be unavailable, making the construction of an IBRB model challenging [17]. Unlike existing methods, DD-IBRB starts from the model structure and achieves the automatic acquisition of the complete IBRB structure from raw data. This includes the construction of reference intervals and the generation of belief degrees, thereby significantly reducing the reliance on expert knowledge. This model can further enhance the modeling capability of the IBRB framework even when expert knowledge is insufficient. In the experimental section, DD-IBRB was compared with other models such as BPNN and LSTM, and the model demonstrated good performance.

**Table 2:** Reference intervals.

No.	Ambient Temperature	Irradiance	Module Temperature	Voltage
1	[0, 0.0789]	[0, 0.0706]	[0, 0.0847]	[0, 0.1071]
2	[0.0789, 0.1580]	[0.0706, 0.1557]	[0.0847, 0.1559]	[0.1071, 0.2183]
3	[0.1580, 0.2219]	[0.1557, 0.2386]	[0.1559, 0.2125]	[0.2183, 0.3126]
4	[0.2219, 0.2806]	[0.2386, 0.3178]	[0.2125, 0.2650]	[0.3126, 0.3928]
5	[0.2806, 0.3350]	[0.3178, 0.3929]	[0.2650, 0.3166]	[0.3928, 0.4666]
6	[0.3350, 0.3844]	[0.3929, 0.4622]	[0.3166, 0.3678]	[0.4666, 0.5375]
7	[0.3844, 0.4304]	[0.4622, 0.5295]	[0.3678, 0.4167]	[0.5375, 0.5995]
8	[0.4304, 0.4735]	[0.5295, 0.5959]	[0.4167, 0.4634]	[0.5995, 0.6548]
9	[0.4735, 0.5192]	[0.5959, 0.6613]	[0.4634, 0.5124]	[0.6548, 0.7085]
10	[0.5192, 0.5712]	[0.6613, 0.7221]	[0.5124, 0.5632]	[0.7085, 0.7601]
11	[0.5712, 0.6273]	[0.7221, 0.7748]	[0.5632, 0.6136]	[0.7601, 0.8050]
12	[0.6273, 0.6835]	[0.7748, 0.8232]	[0.6136, 0.6692]	[0.8050, 0.8460]
13	[0.6835, 0.7435]	[0.8232, 0.8740]	[0.6692, 0.7294]	[0.8460, 0.8872]
14	[0.7435, 0.8263]	[0.8740, 0.9307]	[0.7294, 0.8087]	[0.8872, 0.9358]
15	[0.8263, 1.0000]	[0.9307, 1.0000]	[0.8087, 1.0000]	[0.9358, 1.0000]

**Table 3:** PV power generation capacity grades and reference values.

Reference Degree	D1	D2	D3	D4	D5	D6
Referential value	0	0.13	0.41	0.64	0.82	1

**Table 4:** Initial model for PV power prediction.

No.	Reference Interval	Representative Point	Reliability	Weight	Output
1	[0, 0.0789]	0.0417	1	1	{0.0914, 0.3018, 0.4366, 0.1542, 0.0160, 0.0000}
2	[0.0789, 0.1580]	0.1265	1	1	{0.0765, 0.2733, 0.3080, 0.2700, 0.0670, 0.0052}
3	[0.1580, 0.2219]	0.1915	1	1	{0.0673, 0.1942, 0.3442, 0.2473, 0.1309, 0.0161}
4	[0.2219, 0.2806]	0.2520	1	1	{0.0812, 0.2161, 0.2318, 0.3244, 0.1366, 0.0099}
5	[0.2806, 0.3350]	0.3093	1	1	{0.0406, 0.1467, 0.2644, 0.3517, 0.1747, 0.0219}
6	[0.3350, 0.3844]	0.3626	1	1	{0.0328, 0.1146, 0.2438, 0.3801, 0.2068, 0.0219}
7	[0.3844, 0.4304]	0.4088	1	1	{0.0270, 0.1220, 0.2199, 0.3764, 0.2261, 0.0286}
8	[0.4304, 0.4735]	0.4533	1	1	{0.0394, 0.1356, 0.2065, 0.3340, 0.2630, 0.0215}
9	[0.4735, 0.5192]	0.4946	1	1	{0.0221, 0.0669, 0.1998, 0.4346, 0.2452, 0.0314}
10	[0.5192, 0.5712]	0.5417	1	1	{0.0416, 0.1417, 0.1881, 0.3695, 0.2201, 0.0390}
…	…	…	…	…	…
60	[0.9358, 1.0000]	0.9566	1	1	{0.0000, 0.0000, 0.0000, 0.0000, 0.4926, 0.5074}

**Table 5:** Optimization parameter table.

No.	Reference Interval	Representative Point	Reliability	Weight	Output
1	[0, 0.0789]	0.0417	0.8145	0.5919	{0.0356, 0.3251, 0.3688, 0.2198, 0.0105, 0.0402}
2	[0.0789, 0.1580]	0.1265	0.5000	0.6527	{0.0654, 0.2072, 0.2710, 0.3104, 0.1003, 0.0457}
3	[0.1580, 0.2219]	0.1915	0.6654	0.9972	{0.0398, 0.1252, 0.2634, 0.2876, 0.2013, 0.0827}
4	[0.2219, 0.2806]	0.2520	0.6430	0.7336	{0.0136, 0.2811, 0.1747, 0.2877, 0.1647, 0.0782}
5	[0.2806, 0.3350]	0.3093	0.6398	0.8116	{0.0782, 0.1074, 0.1642, 0.3608, 0.2031, 0.0863}
6	[0.3350, 0.3844]	0.3626	0.5902	0.6531	{0.0913, 0.0691, 0.2069, 0.3670, 0.1937, 0.0720}
7	[0.3844, 0.4304]	0.4088	0.6756	0.6741	{0.0950, 0.0911, 0.1546, 0.3793, 0.2416, 0.0384}
8	[0.4304, 0.4735]	0.4533	0.6154	0.5697	{0.0727, 0.0926, 0.2833, 0.3182, 0.2079, 0.0253}
9	[0.4735, 0.5192]	0.4946	0.7712	0.7320	{0.0501, 0.0692, 0.2306, 0.3543, 0.1928, 0.1030}
10	[0.5192, 0.5712]	0.5417	0.5546	0.9629	{0.0207, 0.0903, 0.1951, 0.3907, 0.2386, 0.0646}
…	…	…	…	…	…
60	[0.9358, 1.0000]	0.9566	1.0000	0.9376	{0.0001, 0.0001, 0.0002, 0.0003, 0.3445, 0.6548}

**Table 6:** Different PV power prediction model.

Model	MSE	SD (MSE)	MAE	$R^{2}$	SMAPE
DD-IBRB	0.00056	0.00001	0.019	0.99	6.01%
DD-IBRB1	0.00270	0	0.041	0.95	12.09%
IBRB	0.00446	0.00029	0.045	0.92	14.24%
IBRB0	0.02280	0	0.094	0.61	24.82%

**Table 7:** PV power prediction model with different proportions.

Training and Testing Ratios	MSE	SD (MSE)	MAE	$R^{2}$	SMAPE
1:2	0.00130	0.00020	0.022	0.97	6.28%
1:1	0.00083	0.00007	0.020	0.98	5.99%
2:1	0.00056	0.00001	0.019	0.99	6.01%
3:1	0.00053	0.00001	0.018	0.99	6.01%

**Table 8:** Comparison of different optimization algorithms.

Model	MSE	SD (MSE)	MAE	$R^{2}$	SMAPE
DD-IBRB (MEAOO)	0.00056	0.00001	0.019	0.99	6.01%
DD-IBRB (AOO)	0.00086	0.00011	0.022	0.98	7.19%
DD-IBRB (P-CMA-ES)	0.00091	0.00004	0.023	0.98	7.79%
DD-IBRB (GA)	0.00119	0.00005	0.027	0.98	8.88%
DD-IBRB (PSO)	0.00155	0.00015	0.029	0.97	9.56%

**Table 9:** Comparison with other BRB models under different training-to-test ratios.

Ratio	Model	MSE	SD (MSE)	MAE	$R^{2}$	SMAPE	*p*-Value
1:2	DD-IBRB	0.00130	0.00020	0.022	0.97	6.28%	/
	IBRB	0.01140	0.00278	0.063	0.81	16.83%	<0.001
	IBRB1	0.01040	0.00161	0.064	0.81	16.63%	<0.001
1:1	DD-IBRB	0.00083	0.00007	0.020	0.98	5.99%	/
	IBRB	0.00790	0.00150	0.057	0.87	15.18%	<0.001
	IBRB1	0.00760	0.00090	0.055	0.88	15.19%	<0.001
2:1	DD-IBRB	0.00056	0.00001	0.019	0.99	6.01%	/
	IBRB	0.00446	0.00029	0.045	0.92	14.24%	<0.001
	IBRB1	0.00404	0.00027	0.043	0.93	13.54%	<0.001

**Table 12:** Comparison with other models.

PART	Model	MSE	SD (MSE)	MAE	$R^{2}$	SMAPE
Part I	DD-IBRB (MEAOO)	0.00102	0.00005	0.022	0.987	9.28%
	IBRB (MEAOO)	0.00441	0.00017	0.045	0.960	17.47%
	DD-IBRB (AOO)	0.00137	0.00008	0.024	0.982	9.77%
	DD-IBRB (GA)	0.00179	0.00008	0.026	0.977	10.53%
	DD-IBRB (P-CAM-ES)	0.00111	0.00008	0.023	0.985	9.26%
Part II	BPNN	0.00150	0.00044	0.026	0.981	11.26%
	SVR	0.00146	0.00013	0.030	0.981	12.82%
	RF	0.00331	0.00062	0.042	0.958	16.45%
	AdaBoost	0.00247	0.00005	0.035	0.968	15.37%

## Data Availability

Data derived from public domain resources.

## Appendix A

**Table A1:** Dictionary of notations.

Notation	Meaning
I	reference intervals of the antecedent attributes
β	belief degrees
x	input data
E(⋅)	function used to generate the reference intervals
ϖ	the set of parameters required for generating the reference intervals
F(⋅)	function used to generate the belief degrees
ϕ	the set of parameters required for generating the belief degrees.
y	PV power prediction result
f(⋅)	the inference function
Ω	model parameters
$\Omega_{best}$	the set of optimized model parameters
Optimize(⋅)	optimization function
τ	the set of parameters required for the optimization process
$x_{i}(i=1,2,\ldots ,M)$	antecedent attributes of the model
M	number of antecedent attributes
$\left\{\left(D_{1},\beta_{1,k}\right),\left(D_{2},\beta_{2,k}\right),\ldots \left(D_{N},\beta_{N,k}\right)\right\}$	belief distribution of the DD-IBRB output results
$D_{i}\left(i=1,\ldots ,N\right)$	ith output grade
N	total number of output grades
$\beta_{i,k}\left(i=1,\ldots ,N\right)$	belief degree corresponding to the ith output grade
$\left[a_{i},b_{i}\right]$	reference interval
L	total number of rules
$w_{k}\left(k=1,\ldots ,L\right)$	weight of the rule
$r_{k}\left(k=1,\ldots ,L\right)$	reliability of the rule
$I_{k}^{R}$	representative point of each reference interval
K	maximum number of clusters K
δ	fuzzification index
W	total number of train samples
$U_{i,j}$	membership degree of the ith data point belonging to the jth cluster
$u_{j}$	cluster center
ς	predefined threshold
T	maximum number of iterations
t	current iteration number
$K_{optimal}$	optimal number of clusters
$I_{j}$	the jth reference interval
$\rho_{i}$	local density of $x_{i}$
$x_{k}$	other data points within the interval
$\left|x_{i}-x_{k}\right|$	absolute distance between two points
γ	controls the smoothness of the exponential kernel
$V_{i}$	absolute distance from $x_{i}$ to the cluster center $u_{j}$.
$\sigma_{j}$	standard deviation of the reference interval
$S_{i}$	overall score
$h_{k}$	rule matching degree
k	represents the rule activated by $x_{i}$
$\gamma_{i}^{k}$	the matching degree between the input sample and the belief rules
$\theta_{i}^{k}$	the activation weight of the rule for sample $x_{i}$
ψ	minimum activation weight preset by the experts
$e_{i}(i=1,\ldots ,L)$	L pieces of independent evidence
Θ	frame of discernment
${\tilde{m}}_{n,i}$	combined probability mass
$u(D_{n})$	utility value of grade $D_{n}$
WT	represents the amount of test data
P	population size
α	disturbance coefficient
ub,lb	upper and lower bounds of the parameter constraints
Ω	population
m	seed mass
l	main awn
ξ	eccentric rotation coefficient
κ	dynamic adjustment factor
q	random number
dim	population dimension
$\Omega_{best}$	best population
G	coefficient vector
g	gravitational acceleration
H	ejection vector
Y	dynamically adjusted range coefficient
ϑ	elasticity coefficient
Δl	variation in the main awn length
∡	ejection angle
ι	air resistance coefficient
Z	random disturbance
$predict(x_{i})$	model’s predicted value
$actual(x_{i})$	true value
$error(x_{i})$	resulting error
PV	photovoltaic
BRB	belief rule base
DD-IBRB	data-driven interval construction belief rule base
GIBM	Gaussian membership interval function
MEAOO	multi-population evolution animated oat optimization
ER	evidential reasoning rules
FCM	fuzzy C-means clustering
BIC	Bayesian information criterion
DWDS	density-weighted distance selection algorithm

**Table A2:** Parameters of the classic models.

Method	Parameters
BPNN	Epochs: 300, Learning rate: 0.01
SVR	Penalty parameter: 1, Epsilon: 0.08
RF	n_estimators: 100, max_features: 2
AdaBoost	n_estimators: 50, min samples leaf: 5
Transformer	num_layers: 2, dropout: 0.1, num_heads: 8
LSTM	Epochs: 300, two LSTM layers (256 and 128 hidden units)
